# The Factors Influencing Depression Endpoints Research (FINDER) study: final results of Italian patients with depression

**DOI:** 10.1186/1744-859X-9-33

**Published:** 2010-07-29

**Authors:** Rosangela Caruso, Andrea Rossi, Alessandra Barraco, Deborah Quail, Luigi Grassi

**Affiliations:** 1 Section of Psychiatry, Department of Medical Sciences of Communication and Behaviour, University of Ferrara, Italy; 2 Medical Department, Eli Lilly Italia, Sesto Fiorentino, Italy; 3 Eli Lilly and Company Limited, Lilly Research Centre, Windlesham, UK; 4 Integrated Department of Mental Health and Drug abuse, NHS Local Health Agency, Ferrara, Italy

## Abstract

**Background:**

Factors Influencing Depression Endpoints Research (FINDER) is a 6-month, prospective, observational study carried out in 12 European countries aimed at investigating health-related quality of life (HRQoL) in outpatients receiving treatment for a first or new depressive episode. The Italian HRQoL data at 6 months is described in this report, and the factors associated with HRQoL changes were determined.

**Methods:**

Data were collected at baseline, 3 and 6 months of treatment. HRQoL was measured using components of the 36-item Short Form Health Survey (SF-36; mental component summary (MCS), physical component summary (PCS)) and the European Quality of Life-5 Dimensions (EQ-5D; visual analogue scale (VAS) and health status index (HSI)). The Hospital Anxiety and Depression Scale (HADS) was adopted to evaluate depressive symptoms, while somatic and painful physical symptoms were assessed by using the 28-item Somatic Symptom Inventory (SSI-28) and a VAS.

**Results:**

Of the initial 513 patients, 472 completed the 3-month observation and 466 the 6-month observation. The SF-36 and EQ-5D mean (± SD) scores showed HRQoL improvements at 3 months and a further smaller improvement at 6 months, with the most positive effects for SF-36 MCS (baseline 22.0 ± 9.2, 3 months 34.6 ± 10.0; 6 months 39.3 ± 9.5) and EQ-5D HSI (baseline 0.4 ± 0.3; 3 months 0.7 ± 0.3; 6 months 0.7 ± 0.2). Depression and anxiety symptoms (HADS-D mean at baseline 13.3 ± 4.2; HADS-A mean at baseline 12.2 ± 3.9) consistently decreased during the first 3 months (8.7 ± 4.3; 7.5 ± 3.6) and showed a further positive change at 6 months (6.9 ± 4.3; 5.8 ± 3.4). Somatic and painful symptoms (SSI and VAS) significantly decreased, with the most positive changes in the SSI-28 somatic item (mean at baseline 2.4 ± 0.7; mean change at 3 months: -0.5; 95% CI -0.6 to -0.5; mean change at 6 months: -0.7; 95% CI -0.8 to -0.7); in 'interference of overall pain with daily activities' (mean at baseline 45.2 ± 30.7; mean change at 3 months -17.4; 95% CI -20.0 to -14.8; mean change at 6 months -24.4; 95% CI -27.3 to -21.6) and in 'having pain while awake' (mean at baseline 41.1 ± 29.0; mean change at 3 months -13.7; 95% CI -15.9 to -11.5; mean change at 6 months -20.2; 95% CI -22.8 to -17.5) domains. The results from linear regression analyses showed that the antidepressant switch within classes was consistently associated with a worsening in SF-36 MCS, EQ-5D VAS and HSI compared to non-switching treatment. Furthermore, between-group antidepressants (AD) switch was associated with a worse SF-36 MCS and EQ-5D HSI. MCS (*P *= 0.028), PCS (*P *= 0.036) and HSI (*P *= 0.002) were inversely related to the number of each previous additional depressive episode. PCS (*P *= 0.009) and HSI (*P *= 0.005) were also less improved in patients suffering from a chronic medical condition. Moreover, PCS (*P *= 0.044) and EQ-5D VAS (*P *< 0.0001) worsening was consistently associated with the presence of a psychiatric illness in the 24 months before baseline. For every additional point on the SSI-somatic score and on the overall pain VAS score at baseline, HSI score were on average 0.062 (*P *< 0.001) and 0.001 (*P *= 0.005) smaller, respectively.

**Conclusions:**

After starting AD treatment, HRQoL improvements at 3 and 6 months were observed. However, several factors can negatively influence HRQoL, such as the presence of somatic and painful symptoms, the presence of any chronic medical condition or previous psychiatric illness.

## Background

The role of depression, a common and debilitating condition, in negatively influencing the quality of life of patients such as functioning by affecting psychological, physical and social areas of life has been the object of intense research over the last few years [1]. Several studies have investigated these aspects in recent years. A large study of 25,916 primary care patients from several countries revealed that patients with major depression reported higher levels of disability than those without depression [2]. In a different study carried out in Europe, Lepine *et al*. [3] found that the degree of disability was related to severity of depression in patients with major depressive disorder (MDD).

Goldney *et al*. [4], by studying a large sample of the Australian population, found that all the dimensions of quality of life, as measured by the 36-item Medical Outcome Short-Form Health Survey (SF-36), were poorer among patients with depression (n = 319) with respect to the non-depressed general population (n = 2,486), with the poorest level reached by patients with MDD (n = 205). Data indicating that the severity of depression was significant in negatively influencing the quality of life of patients has been confirmed by Trompenaars *et al*. [5], who showed lower levels of quality of life among patients with major depression with respect to those with dysthymia and adjustment disorders.

A large population-based study involving 60 countries has also indicated that depression had the largest effect on worsening mean health scores compared with other chronic conditions, including physical diseases, and that patients with depression comorbid with one or more chronic diseases had the worst health scores of all the disease states [6].

Somatic symptoms, including pain, are a specific component of depression, and were studied as a factor influencing the quality of life of patients. In a recent Spanish study involving 1,150 primary care patients with a *Diagnostic and Statistical Manual of Mental Disorders, fourth edition *(DSM-IV)-defined diagnosis of MDD, García-Campayo *et al*. [7] found that somatic symptom-associated disability and a number of somatic symptoms are strongly associated with increased depression severity and health resource use, as well as with decreased quality of life (QOL).

Other factors that may have a great influence on the outcome of a depressive episode, namely the patient's psychiatric and medical history, the presence of somatic symptoms, including painful symptoms, and demographic and social variables, need to be studied in a prospective way.

Recent results have been reported by the European Union Factors Influencing Depression Endpoint Research (EUFINDER), a 6-month, prospective, observational study carried out in 12 European countries, aimed at assessing the changes of health-related quality of life (HRQoL) in outpatients receiving pharmacological treatment for a depressive episode in routine primary care and specialist settings. The study, carried out on 3,468 adult patients with a clinically diagnosed episode of depression, seems to demonstrate that receiving an antidepressant (AD) treatment was associated with improvements in HRQoL, as assessed by the 36-item Short Form Health Survey (SF-36) and the European Quality of Life-5 Dimensions (EQ-5D) [8-10]. The baseline results of patients in the FINDER study in Italy, enrolled only by specialists, have confirmed a clinically significant association of depression with clinical and functional impairments, as well as poor HRQoL [11]. The aim of the present report is to describe the Italian HRQoL data at 6 months after starting pharmacotherapy for depression, and to determine what factors are associated with HRQoL changes over time.

## Methods

### Study design and subjects

The design and methods of FINDER have been reported in detail elsewhere [8,9]. Briefly, primary care physicians or specialists enrolled adult patients responding to the following inclusion criteria: (i) A clinical diagnosis of depression; (ii) pharmacological treatment required for either a first or a new episode of depression; (iii) at least 18 years of age; (iv) no simultaneous participation in a different study that included an investigational drug or procedure.

All treatment choices and follow-up care were at the discretion of the physician and the patient according to the physician's clinical judgment and the local standard of medical care. The study was approved according to requirements for ethics and all patients gave written informed consent. Following the baseline visit, data were recorded after approximately 3 and 6 months of therapy, during routine care visits.

### Data collected

At baseline, patient sociodemographic data were recorded, along with psychiatric history, duration of the current depressive episode, drugs used in the last 24 months and prescribed at enrolment, and presence of medical comorbidities. Antidepressants prescribed were grouped as follows: selective serotonin reuptake inhibitors (SSRIs: citalopram, escitalopram, fluoxetine, fluvoxamine, paroxetine, sertraline); serotonin noradrenaline reuptake inhibitors (SNRIs: venlafaxine); tricyclic and tetracyclic antidepressants (TCAs: amitriptyline, clomipramine, imipramine, maprotiline, mianserine, nortriptyline), others (mirtazapine, phenelzine, reboxetine, trazodone, lithium), or combinations of antidepressants (Table 1).

**Table 1:** Medications for depression prescribed at enrolment

SSRI, drugs only, n = 331 (64.5%)	SNRI, drugs only, n = 76 (14.8%)	TCA, drugs only, n = 33 (6.4%)	Other drugs, n = 41 (8.0%)	Combinations, n = 32(6.2%)
Citalopram	Venlafaxine	Amytriptyline	Mirtazapine	
Escitalopram		Clomipramine	Phenelzine	
Fluoxetine		Imipramine	Reboxetine	
Fluvoxamine		Maprotiline	Trazodone	
Paroxetine		Mianserin	Lithium	
Sertraline		Nortriptyline		

SSRI = selective serotonin reuptake inhibitors; SNRI = serotonin noradrenaline reuptake inhibitors; TCA = tricyclic/tetracyclic antidepressants.

### Instruments

HRQoL was measured using the SF-36 version 2.0 [12] and the EQ-5D [13]. Severity of anxiety and depression symptoms were scored using the Hospital Anxiety and Depression Scale (HADS) subscales: HADS-D (depression) and HADS-A (anxiety) [14]. Somatic and painful symptoms were assessed by using the 28-item Somatic Symptom Inventory (SSI-28) [15] and a visual analogue scale (VAS, 0-100) evaluating overall pain severity. Details regarding the instrument's use are provided elsewhere [9].

### Statistical analysis

Descriptive statistics (mean + SD for continuous variables; percentage for categorical variables) were calculated for each variable to describe characteristics of all 513 patients at baseline. However, only those with at least one follow-up visit were included in the regression analyses. All confidence intervals were calculated at the 95% level.

Patients were excluded from the analysis if one or more entry criteria were violated or from individual analyses based on missing, implausible (according to predefined ranges) or uninterpretable data. The exclusion of patient data from the analysis was based on study entry criteria at the first observation, not on whether or not antidepressant medication was taken during the 6-month follow-up period. Only one patient was excluded from the analysis (0.2%) due to failure to meet entry criteria. Data were analysed using SAS V.8.2 (SAS, Cary, NC, USA).

### Loss to follow-up analysis

No predictive analysis was performed for the Italian subset. Details of the analysis performed for the EUFINDER study were previously described by Reed *et al*. [10].

### Main analysis

Backward regression analysis was performed to identify variables independently associated with HRQoL outcomes. Separate models were fitted for each of the following outcome variables: SF-36 (mental component summary (MCS), physical component summary (PCS)), EQ-5D (VAS, health status index (HSI)). A mixed effects model repeated measures (MMRM) analysis with unstructured covariance structure was used. Independent variables were removed from the full model until only statistically significant (*P ***≤ **0.05) variables remained.

Independent variables in the model included both baseline as well as post-baseline covariates: score for the dependent variable at baseline, age, gender, education (2 levels: none/mandatory, further), occupational status (3 levels: 'working for pay', 'unemployed', 'other'), marital status (married/domestic partner vs other), body mass index (BMI; continuous), number of dependants, smoking (yes/no), number of previous episodes of depression, age at first episode, any psychiatric illnesses in the 24 months before baseline (yes/no), duration (at baseline) of the current MDD episode (continuous), HADS anxiety score (continuous), HADS depression score (continuous), overall pain VAS score at baseline, SSI-somatic score at baseline, any chronic medical conditions (yes/no), class of antidepressant taken between baseline and 6-month observation, switch of antidepressant after 3 months of treatment (within AD class, between AD classes, without AD change). No interaction terms were included.

## Results

Sociodemographic data, psychiatric history and medical comorbidity

Psychiatrists operating in specialist settings enrolled 513 patients from 38 Italian centres. Sociodemographic and clinical data with the patients' characteristics are reported elsewhere, with all the baseline details of the Italian study [11]. In summary, all patients (139 males, 27.1% and 373 females, 72.9%; mean ± SD age 49.2 ± 15.2 years) already had a clinical depression diagnosis. Approximately half of patients were married and 34.4% were employed. A total of 302 patients (58.9%) had received mandatory or no education and 211 (41.1%) had received further education. The mean (± SD) duration of depression was 10.6 ± 12.3 years and the mean (± SD) age at the first depressive episode was 38.7 ± 15.9 years. Approximately half of patients reported at least one episode of depression in the 24 months before baseline. Anxiety/panic disorders were the most common psychiatric comorbidities in the last 24 months and were reported in 72.6% of patients, while 35.9% of patients had functional somatic syndromes: chronic fatigue syndrome (17.1%), irritable bowel syndrome (16.6%) and atypical chest pain (11.2%) were the most common ones. Approximately half of the patients reported other non-psychiatric diseases: arterial hypertension (25.4%) and rheumatological disorders (16.2%) were the most common conditions indicated by physicians from a checklist of disorders. In all, 20.3% of patients reported significant pain (VAS score for overall pain at baseline > 30 mm is considered significant or moderate/severe) and had a defined medical disorder known to cause pain, while 44.9% reported significant pain without a medical disorder known to cause it.

The antidepressants prescribed in at least 10% of patients were: sertraline (89 patients, 17.3%), escitalopram (83, 16.2%), venlaflaxine (80, 15.6%), paroxetine (76, 14.8%) and citalopram (62, 12.1%) (Table 1).

Of the initial 513 patients, 472 completed the 3-month observation (92.0%), and 466 completed the 6-month observation (90.8%).

### HRQoL and clinical changes over time

The mean scores for SF-36 and EQ-5D, at baseline, 3 and 6 months indicated HRQoL improvements at 3 months and further smaller improvement at 6 months, with the most positive effects observed for SF-36 MCS and EQ-5D HSI. Depression and anxiety symptoms, scored by using HADS-D and HADS-A subscales consistently decreased during the first 3 months and showed a further positive change at 6 months (Table 2). Somatic and painful symptoms, as measured by using SSI and VAS outcomes, showed significant decreases over time, with the most positive changes observed for SSI-28 Somatic Item and, as far as VAS is concerned, for 'interference of overall pain with daily activities' and 'having pain while awake' domains (Tables 3 and 4).

**Table 2:** SF-36, EQ-5D and HADS values at baseline, 3 months and 6 months

Variable	Baseline, mean ± SD	3 Months		6 Months	
		
		Mean ± SD	95% CI	Mean ± SD	95% CI
SF-36 MCS	22.0 ± 9.2	34.6 ± 10.0	33.7 to 35.6	39.3 ± 9.5	38.4 to 40.1
SF-36 PCS	44.9 ± 9.7	46.9 ± 8.5	46.1 to 47.7	49.0 ± 8.5	48.2 to 49.8
EQ-5D HSI	0.40 ± 0.01	0.66 ± 0.26	0.63 to 0.68	0.74 ± 0.23	0.72 to 0.76
EQ-5D VAS	45.7 ± 19.6	61.3 ± 17.9	59.7 to 63.0	69.3 ± 17.0	67.8 to 70.9
HADS-D	13.3 ± 4.2	8.7 ± 4.3	8.3 to 9.1	6.9 ± 4.3	6.5 to 7.3
HADS-A	12.2 ± 3.9	7.5 ± 3.6	7.2 to 7.8	5.8 ± 3.4	5.5 to 6.1

EQ-5D = European Quality of Life-5 Dimensions; HADS-A/D = Hospital Anxiety and Depression Scale - anxiety/depression; HSI = health status index; MCS = mental component summary; PCS = physical component summary; SF-36 = Short Form 36; VAS = visual analogue scale.

**Table 3:** Summary statistics for change from baseline in VAS (mm) at 3-month and 6-month observations

Observation		N	Mean	Median	SD	95% CI
Baseline	Overall VAS score	479	42.9	45.0	27.1	41.7 to 44.1
	Severity of headaches during the past week	476	31.5	25.5	28.3	30.2 to 32.8
	Severity of back pain during the past week	476	32.8	30.0	28.6	31.5 to 34.1
	Severity of shoulder pain during the past week	478	29.7	20.0	29.5	28.4 to 31.0
	Interference of overall pain(s) with ability to do daily activities during the past week	477	45.2	47.0	30.7	43.8 to 46.6
	Having pain(s) while awake during the past week	479	41.1	40.0	29.0	39.8 to 42.4
3 months	Change in severity of overall pain(s) during the past week	420	-12.9	-10.0	23.1	-15.1 to -10.7
	Change in severity of headaches during the past week	417	-9.5	-7.0	24.1	-11.8 to -7.1
	Change in severity of back pain during the past week	416	-5.3	-2.0	23.8	-7.6 to -3.0
	Change in severity of shoulder pain during the past week	420	-4.9	0.0	25.0	-7.3 to -2.5
	Change in interference of overall pain(s) with ability to do daily activities during the past week	419	-17.4	-12.0	27.5	-20-0 to -14.8
	Change in having pain(s), while awake during the past week	419	-13.7	-10.0	23.2	-15.9 to -11.5
6 months	Change in severity of overall pain(s) during the past week	421	-20.1	-19.0	27.1	-22.7 to -17.5
	Change in severity of headaches during the past week	421	-14.5	-10.0	28.0	-17.2 to -11.9
	Change in severity of back pain during the past week	419	-10.3	-5.0	27.3	-12.9 to -7.7
	Change in severity of shoulder pain during the past week	420	-10.1	-5.0	27.1	-12.7 to -7.5
	Change in interference of overall pain(s) with ability to do daily activities during the past week	420	-24.4	-21.0	30.1	-27.3 to -21.6
	Change in having pain(s), while awake during the past week	422	-20.2	-19.0	28.0	-22.8 to -17.5

VAS = visual analogue scale.

**Table 4:** Summary statistics for change from baseline in SSI-28 at 3-month and 6-month observations

Observation		N	Mean	Median	SD	95% CI
Baseline	Summary of SSI-28 mean score	510	2.4	2.3	0.7	2.3 to 2.4
	Summary of SSI-28 pain item mean score	498	2.3	2.1	0.8	2.3 to 2.4
	Summary of SSI-28 somatic item mean score	511	2.4	2.3	0.7	2.4 to 2.4
3 months	Change in SSI-28 mean score	465	0.5	0.4	0.5	0.5 to 0.4
	Change in SSI-28 pain item mean score	447	-0.3	-0.3	0.6	-0.4 to -0.3
	Change in SSI-28 somatic item mean score	466	-0.5	-0.5	0.6	-0.6 to -0.5
6 months	Change in SSI-28 mean score	461	-0.7	-0.6	0.6	-0.7 to -0.6
	Change in SSI-28 pain item mean score	441	-0.5	-0.4	0.7	-0.6 to -0.4
	Change in SSI-28 somatic item mean score	461	-0.7	-0.7	0.6	-0.8 to -0.7

SSI-28 = 28-item Somatic Symptom Inventory.

### Factors associated with HRQoL changes over 6 months

#### SF-36 MCS

The analysis was performed on a total of 404 patients having a total of 797 post-baseline observations (see Table 5). Baseline MCS value was associated with MCS score through the study with on average scores of MCS 0.35 points greater (*P *< 0.0001; 95% CI 0.27 to 0.44) for each additional point on the scale at baseline. The MCS component of SF 36 MCS was significantly associated with the switch of type or class of antidepressant between the first and second 3-month periods of the study when compared with no switch during the study (*P *< 0.0001). When the switch was between antidepressants belonging to different classes, the average effect observed with the switch to a different class of AD was -3.39 points compared to no switch (95% CI -6.13 to -0.65), while the effect when the switch was to an AD within the same therapeutic class was -8.50 (95% CI -12.46 to -4.54). MCS was also less improved in patients who were unemployed (estimate = -4.45; *P *= 0.0005; 95% CI -6.96 to -1.94) or with 'other' employment status (estimate = -3.23; *P *= 0.0002; 95% CI -4.92 to -1.54) compared to working for pay.

**Table 5:** Factors significantly associated with SF-36 MCS over 6 months of AD treatment

Independent variables	F, *P *value	Estimate
MCS at baseline	64, < 0.0001	0.35
Switch within or between AD class (2 df) (reference no switch)	11, < 0.0001	Between -3.39, within -8.50
Occupational status (2 df, reference working for pay)	9, < 0.001	Other -3.23, unemployed -4.45
No. of previous episodes of depression	5, 0.028	-0.68

No. of patients = 404, no. of observations = 797. Switch = change between what was taken between baseline and 3-month observation and what was taken between the 3-month and 6-month observations.

AD = antidepressants; df = degrees of freedom; MCS = mental component summary; SF-36 = Short Form 36.

Finally, the number of previous episodes of depression was significantly associated with MCS score, with each additional episode associated with an MCS score -0.68 points lower (*P *= 0.028; 95% CI -1.29 to -0.07).

#### SF-36 PCS

The analysis was performed on 371 patients, with a total of 716 post-baseline observations (Table 6). PCS baseline value was associated with scores through 3-month and 6-month observations with on average scores of PCS 0.47 points greater (*P *< 0.0001; 95% CI 0.40 to 0.54). Older age was statistically related to a smaller improvement of PCS component of SF-36 (estimate = -0.10; *P *< 0.0001; 95% CI -0.14 to -0.05). PCS outcome was also inversely associated to the number of previous depressive episodes (estimate = -0.47; *P *= 0.036; 95% CI -0.90 to -0.03) and with SSI-somatic score at baseline (estimate = -1.16; *P *= 0.034 95% CI -2.23 to -0.09). This means that patients with a higher baseline SSI somatic score had poorer physical QOL outcomes. PCS was also less improved in patients suffering from a chronic medical condition (estimate = -1.61; *P *= 0.009; 95% CI -2.80 to -0.41) and in patients who presented any psychiatric illnesses in the 24 months before baseline (estimate = -1.42; *P *= 0.044; 95% CI -2.80 to -0.04). Better PCS scores were associated with use of an SNRI (estimate = 3.24; *P *= 0.014; 95% CI 0.66 to 5.82) or 'other drugs' (estimate = 3.82; *P *= 0.018; 95% CI 0.67 to 6.97) baseline and 3-month observation compared to TCA. PCS improvement was also statistically associated with HADS-A score at baseline (estimate = 0.24; *P *= 0.009; 95% CI 0.06 to 0.42;) and with the number of the patient's dependants (estimate = 0.41; *P *= 0.050; 95% CI 0.001 to 0.822).

**Table 6:** Factors significantly associated with SF-36 PCS over 6 months of AD treatment

Independent variables	F, *P *value	Estimate	95% CI
PCS at baseline	172, < 0.0001	0.47	0.40 to 0.54
Age	19, < 0.0001	-0.10	-0.14 to -0.05
Any chronic medical conditions (yes vs no)	7, 0.009	-1.61	-2.80 to -0.41
HADS-A at baseline	7, 0.009	0.24	0.06 to 0.42
SSI-somatic at baseline	5, 0.034	-1.16	-2.23 to -0.09
No. of previous episodes of depression	4, 0.036	-0.47	-0.90 to -0.03
Any psychiatric illnesses in the 24 months before baseline observation	4, 0.044	-1.42	-2.80 to -0.04
Class of AD taken between baseline and 3-month observations (4 df) (reference TCA)	2, 0.045	Combinations (NS)	
		Other drugs 3.82	0.67 to 6.97
		SNRI 3.24	0.66 to 5.82
		SSRI (NS)	
No. of dependants	4, 0.050	0.41	0.001 to 0.822

No. of patients = 371, no. of observations = 716.

AD = antidepressant; df = degrees of freedom; HADS-A = Hospital Anxiety and Depression Scale - anxiety; NS = not significant; PCS = physical component summary; SF-36 = Short Form 36; SSI = Somatic Symptom Inventory; TCA = tricyclic/tetracyclic antidepressants.

#### EQ-5D HSI

The analysis was performed on 328 patients, with a total of 650 post-baseline observations (Table 7). Baseline HSI values were associated with HSI scores over 6 months with on average scores of HSI 0.208 points greater (*P *< 0.0001; 95% CI 0.136 to 0.280) for each additional point on the scale at baseline.

**Table 7:** Factors significantly associated with EQ-5D HSI over 6 months of AD treatment

Independent variables	F, *P *value	Estimate	95% CI
HSI at baseline	32, < 0.0001	0.208	0.136 to 0.280
Switch within or between AD class (2 df) (reference no switch)	11, < 0.0001	Between -0.071 Within -0.222	-0.136 to -0.006 -0.323 to -0.121
SSI-somatic at baseline	12, < 0.001	-0.062	-0.098 to -0.026
No. of previous episodes of depression	9, 0.002	-0.022	-0.036 to -0.008
Any chronic medical conditions (yes vs no)	8, 0.005	-0.055	-0.094 to -0.017
Overall pain VAS at baseline	8, 0.005	-0.001	-0.002 to -0.0003
No. of dependants	7, 0.010	0.018	0.004 to 0.032
HADS-A at baseline	6, 0.019	0.008	0.001 to 0.013

No. of patients = 328, no. of observations = 650. Switch = change between what was taken between baseline and 3-month observation and what was taken between the 3-month and 6-month observations.

AD = antidepressant; df = degrees of freedom; EQ-5D = European Quality of Life-5 Dimensions; HADS-A, Hospital Anxiety and Depression Scale - anxiety; HSI, health status index; SSI = Somatic Symptom Inventory; VAS = visual analogue scale.

A small worsening of HSI scores was associated with the switch to a different antidepressant, when compared to no switch, with some difference in case of switch between two classes of AD (estimate = -0.071; *P *= 0.032; 95% CI -0.136 to -0.006) or within the same class (estimate = -0.222; *P *< 0.0001; 95% CI -0.323 to -0.121). HSI outcomes were inversely related to SSI-somatic scores at baseline (estimate = -0.062; *P *< 0.001; 95% CI -0.098 to -0.026), to the number of previous episodes of depression (estimate = -0.022; *P *= 0.002; 95% CI -0.036 to -0.008) and to overall pain VAS scores at baseline (estimate = -0.001; *P *= 0.005; 95% CI -0.002 to 0.0003). HSI results were also less improved when there was the presence of a chronic medical condition (estimate = -0.055; *P *= 0.005; 95% CI -0.094 to -0.017).

HSI improvement was statistically directly related to HADS-A scores (estimate = 0.008; *P *= 0.019; 95% CI 0.001 to 0.013) at baseline and to the number of the patient's dependants (estimate = 0.018; *P *= 0.010; 95% CI 0.004 to 0.032).

#### EQ-5D VAS

The analysis was performed on 400 patients, with a total of 795 observations (Table 8). Baseline EQ-5D VAS values were associated with EQ-5D VAS through the study with on average scores of 0.33 points greater (*P *< 0.0001; 95% CI 0.26 to 0.40) for each additional point on the scale at baseline.

**Table 8:** Factors significantly associated with EQ-5D VAS over 6 months of AD treatment

Independent variables	F, *P *value	Estimate	95% CI
EQ-5D VAS at baseline	85, < 0.0001	0.33	0.26 to 0.40
Any psychiatric illnesses in the 24 months before baseline	18, < 0.0001	-7.18	-10.53 to -3.84
Switch within or between AD class (2 df) (reference no switch)		9, < 0.001	Between (NS)
		Within -15.51	-22.66 to -8.36
Occupational status (2 df, reference working for pay)	8, < 0.001	Other -4.16	-7.43- to -0.89
		Unemployed -8.46	12.77 to -4.14
Age	13, < 0.001	-0.18	-0.29 to -0.08
Overall pain VAS at baseline	8, 0.004	-0.08	-0.13 to -0.02

No. of patients = 400, no. of observations = 795. Switch = change between what was taken between baseline and 3-month observation and what was taken between the 3-month and 6-month observations.

AD = antidepressant; df = degrees of freedom; EQ-5D = European Quality of Life-5 Dimensions; VAS = visual analogue scale.

However, an EQ-5D VAS worsening was consistently associated with the switch of antidepressant within the same class (estimate = -15.51; *P *< 0.0001; 95% CI -22.66 to -8.36), and with the presence of a psychiatric illness in the 24 months before baseline (estimate = -7.18; *P *< 0.0001; 95% CI -10.53 to -3.84). A patient's occupational status appeared to significantly influence EQ-5D VAS outcomes, as EQ5D VAS was also less improved in unemployed patients (estimate = -8.46; *P *< 0.0001; 95% CI -12.77 to -4.14) and in patients with 'other occupations' (estimate = -4.16; *P *= 0.013; 95% CI -7.43 to -0.89) when compared to patients working for pay.

Older age (estimate = -0.18; *P *= 0.0005; 95% CI -0.29 to -0.08) and overall pain VAS values at baseline (estimate = -0.08; *P *= 0.004; 95% CI -0.13 to -0.02), showed a statistical inverse relation with EQ-5D VAS outcomes (Table 8).

## Discussion

MDD is often associated with an impaired patient biopsychosocial functioning, and with reductions in health-related quality of life. Nonetheless, the importance of assessing MDD influence on HRQoL and the role of MDD clinical symptoms on HRQoL has only recently been recognised [6,16].

A recent European observational study, EUFINDER, carried out in 12 countries, evaluated the changes of HRQoL in outpatients receiving pharmacological treatment for a depressive episode in routine primary care and specialist settings.

Receiving an AD treatment was associated with large improvements in HRQoL, as assessed by the SF-36 and the EQ-5D [8,7,10]. In order to understand in a specific cultural context, such as Italy, the changes in the measures as assessed in the FINDER study, we have confirmed clinical and functional impairments and poor HRQoL in the baseline assessment [11].

In this study we examined the follow-up data on the Italian FINDER sample in order to verify the role of depression on HRQoL and found that patients with a clinical diagnosis of depression experienced improvements in HRQoL after starting an antidepressant treatment. More specifically, several dimensions of HRQoL were shown to improve in a 6-month period, including SF-36, scores, especially mental health scores and EQ-5D HSI.

These results are consistent with a recent longitudinal study [17], which found reduced EQ-5D scores in Swedish primary care patients with depression compared to the general population and comparable HRQoL improvements after 6 months of treatment.

Interestingly, our study also showed that the switch of antidepressant within AD groups was consistently associated with a smaller improvement in SF-36 MCS and EQ-5D VAS and HSI compared to patients not switching treatment. Furthermore, between-group AD switch was associated with a worse SF-36 MCS and EQ-5D HSI compared to no switch. This data may be due to the higher severity of depression in patients requiring a switch of treatment, to an inappropriate diagnosis or to inadequate doses/exposure times or inappropriate exposure time rather than to the switching itself. A second result of our study was that HADS-A scores at baseline were positively associated with HRQoL improvements at 6 months. This might be partially due to a positive effect of participating in the trial, but also to the positive effects of antidepressant treatments on anxiety.

A third finding is that the presence of a previous psychiatric illness in the 24 months before baseline was predictive of poorer HRQoL outcomes, with respect to EQ-5D VAS and, to a lower degree, to SF-36 PCS.

Another result of our study indicates that a higher severity of somatic and painful symptoms at baseline, as evaluated by patients, was associated with poorer HRQoL outcomes during antidepressant treatment. We also found that the presence of a chronic medical disease is associated with poorer HRQoL, as far as SF-36 PCS and EQ-5D HSI are concerned. This confirms other research highlighting the role of somatic and painful symptoms in the QOL of depressed patients and their possible implications on depression outcomes [7,10,18,19].

There are some limitations that need to be considered in interpreting the results of the study presented here. First, important data such as the association between HRQoL outcomes and AD switching need to be further investigated in more controlled settings. A second limitation is that this study did not include a control group, for example a group of patients not starting an AD treatment. As a third limitation, HRQoL measures evaluate concepts also included in instruments that assess depression. However, SF-36 and EQ-5D focus more on the patients' daily living, social interactions and related aspects. Symptom severity and impact on everyday life are probably closely correlated, which may explain much of the parallel improvement in HRQoL and depression symptoms. Furthermore, the broad number of rating scales used in the study limited the practicability of a longer follow-up. This may have in part conditioned findings related to the effects of antidepressant treatment on HRQoL. Lastly, during our observation period, patients did not reach the general population average of 50 for the SF-36 MCS (MCS mean value at 6 months = 39.3). Thus, a longer observation period might be needed to assess whether, for patients with depressive disorders, the time for achieving mental HRQoL outcomes comparable to the general population is longer than 6 months or whether, even after treatment with antidepressants, in these patients HRQoL remains impaired.

## Conclusions

Our study underlined that the presence of somatic and painful symptoms, chronic medical conditions or previous psychiatric illnesses are to be taken into account when offering treatment to a depressed patient, as they can negatively influence HRQoL. Moreover depressed patients with higher levels of somatic and painful symptoms may respond less to treatment than other patients.

A consequent clinical implication of these findings is that specific pharmacological profiles could be considered when prescribing antidepressants (for example, SSRIs and SNRIs) to patients reporting painful physical symptoms.

Furthermore, our study showed that switching between different AD classes was consistently associated with poorer HRQoL outcomes compared to patients able to remain within the same class of AD.

## Competing interests

AB and AR are employees at Eli Lilly Italia.

## Authors' contributions

AR and AB made substantial contributions to analysis and interpretation of data, and in the revision of the manuscript. DQ made substantial contributions to statistical analyses and to the revision of the manuscript. LG made relevant contribution in the recruitment of patients, in the critical revision of the manuscript. RC made substantial contributions in the analysis and interpretation of data and in the manuscript writing. All authors gave final approval for the article.
